# Associations between childhood maltreatment and psychiatric disorders: analysis from electronic health records in Hong Kong

**DOI:** 10.1038/s41398-022-01986-y

**Published:** 2022-06-06

**Authors:** Rosa S. Wong, Keith T. S. Tung, Frederick K. W. Ho, Tatia M. C. Lee, Ko Ling Chan, John Bacon-Shone, David Coghill, Kenneth K. C. Man, Pak C. Sham, Wilfred H. S. Wong, Winnie W. Y. Tso, Gilbert T. Chua, Ian C. K. Wong, Patrick Ip

**Affiliations:** 1 Department of Paediatrics and Adolescent Medicine, The University of Hong Kong, Hong Kong, China; 2 Centre for Safe Medication Practice and Research, Department of Pharmacology and Pharmacy, The University of Hong Kong, Hong Kong, China; 3 Department of Social Work and Social Administration, The University of Hong Kong, Hong Kong, China; 4 Institute of Health and Wellbeing, University of Glasgow, Glasgow, UK; 5 State Key Laboratory of Brain and Cognitive Sciences, The University of Hong Kong, Hong Kong, China; 6 Laboratory of Neuropsychology, The University of Hong Kong, Hong Kong, China; 7 Department of Applied Social Sciences, The Hong Kong Polytechnic University, Hong Kong, China; 8 Social Sciences Research Centre, The University of Hong Kong, Hong Kong, China; 9 Department of Paediatrics and Psychiatry, Faculty of Medicine, Dentistry and Health Sciences, University of Melbourne, Melbourne, VIC Australia; 10 Murdoch Children’s Research Institute, Melbourne, VIC Australia; 11 Research Department of Practice and Policy, UCL School of Pharmacy, London, UK; 12 Department of Psychiatry, The University of Hong Kong, Hong Kong, China; 13 Centre for PanorOmic Sciences, The University of Hong Kong, Hong Kong, China

**Keywords:** Psychiatric disorders, Human behaviour

## Abstract

There has been a lack of high-quality evidence concerning the association between childhood maltreatment and psychiatric diagnoses particularly for Axis II disorders. This study aimed to examine the association between childhood maltreatment exposure and Axis I and Axis II psychiatry disorders using electronic health records. In this study, the exposed group (*n* = 7473) comprised patients aged 0 to 19 years with a first-time record of maltreatment episode between January 1, 2001 and December 31, 2010, whereas the unexposed group (*n* = 26,834) comprised individuals of the same gender and age who were admitted into the same hospital in the same calendar year and month but had no records of maltreatment in the Hong Kong Clinical Data Analysis and Reporting System (CDARS). Data on their psychiatric diagnoses recorded from the date of admission to January 31, 2019 were extracted. A Cox proportional hazard regression model was fitted to estimate the hazard ratio (HR, plus 95% CIs) between childhood maltreatment exposure and psychiatric diagnoses, adjusting for age at index visit, sex, and government welfare recipient status. Results showed that childhood maltreatment exposure was significantly associated with subsequent diagnosis of conduct disorder/ oppositional defiant disorder (adjusted HR, 10.99 [95% CI 6.36, 19.01]), attention deficit hyperactivity disorder (ADHD) (7.28 [5.49, 9.65]), and personality disorders (5.36 [3.78, 7.59]). The risk of psychiatric disorders following childhood maltreatment did not vary by history of childhood sexual abuse, age at maltreatment exposure, and gender. Individuals with a history of childhood maltreatment are vulnerable to psychiatric disorders. Findings support the provision of integrated care within the primary health care setting to address the long-term medical and psychosocial needs of individuals with a history of childhood maltreatment.

## Introduction

Childhood maltreatment, including all forms of abuse and neglect, is a significant public health problem that can have short- and long-term negative effects on the health, education, and wellbeing of children [1]. Evidence from a series of meta-analyses indicates that the pooled global prevalence rates of physical abuse, psychological abuse, sexual abuse, and neglect are 17.7%, 26.7%, 11.8%, and 16.3%, respectively [2–5]. In the United States, more than one third of children (37.4%) have experienced maltreatment at least once by age 18 years [6]. In China, the estimated prevalence is 26.6% for physical abuse, 19.6% for emotional abuse, 8.7% for sexual abuse, and 26.0% for neglect [7]. Although much effort has been made to prevent domestic and family violence, the incidence and prevalence of childhood maltreatment in many countries remain relatively stable [8]. Likewise, childhood maltreatment is also a matter of public concern in Hong Kong, where the hospitalization rate has doubled during the period from 2001 to 2010 [9].

Evidence, mainly from western countries, suggests that childhood maltreatment is associated with an increased risk of physical and mental disorders at older ages. A meta-analysis of 124 studies reported that psychiatric diagnoses such as depressive disorders, anxiety disorders, drug use, and suicide attempts are among the long-term consequences of non-sexual childhood maltreatment (physical abuse, emotional abuse, and neglect) [10]. Another meta-analysis of cross-sectional studies in China found that children exposed to physical abuse had more than twice the odds of psychiatry disorders compared to those without exposure [11]. However, many of the included studies had methodological limitations such as cross-sectional design, use of self-reported childhood maltreatment questionnaires, small sample size, and lack of standardized tools to assess mental health outcomes [1, 10, 11]. These limitations may have introduced bias in data analysis, resulting in an inaccurate estimate of the association between childhood maltreatment and psychiatry morbidity later in life. In western countries, although findings from prospective studies have been reported, mental health outcomes in these studies were often assessed using self-report or proxy-report measures [10]. Where clinical data have been used, only early-onset symptoms [12] and more common psychiatric disorders such as depression and anxiety disorders [1] were examined. Evidence to support the risk of other psychiatric disorders such as bipolar disorder and psychosis following childhood maltreatment is still scant. Notably, there has been a limited body of research on this topic using hospital administrative data in non-western populations.

Apart from methodological issues, given that some children can continue to thrive in the face of adversity, the long-term consequences of childhood maltreatment may depend on a range of individual and contextual factors. Examination of these factors would not only help to reveal the optimal timing for intervention but also help to identify the characteristics of the vulnerable groups. In addition, the findings can inform guidelines for setting up priorities in allocation of limited healthcare resources to improve the long-term health outcomes of child maltreatment victims. Previous studies have demonstrated that the manifestation of psychiatric disorders after adverse events is subject to a range of personal and event factors such as gender, age of onset of child maltreatment, and type of maltreatment exposure during childhood. For example, gender differences in mental health outcomes have received mixed evidence [13, 14]. A small yet growing body of survey research suggests that early-life maltreatment experiences, in particular with those occurring during the first 5 years of life, are associated with a multitude of developmental problems [15]. However, little research has been done to investigate the role of age of onset of child maltreatment in the prediction of psychiatric disorders at older ages. Furthermore, in spite of the notable increase in the rate of hospitalization for childhood sexual abuse in Hong Kong [9], the association between childhood sexual abuse and later psychopathology has been underexplored in non-western populations.

Hence, this population-based cohort study has two objectives. The first is to investigate the association of child maltreatment events occurring between 2001 and 2010 with a broad range of psychiatric diagnoses using data from the territory-wide hospital administrative database in Hong Kong. The second objective is to examine whether the strength of association would vary significantly by gender, age of onset, and history of childhood sexual abuse. We hypothesized that individuals exposed to childhood maltreatment were more likely than their non-exposed counterparts to develop psychiatric disorders at older ages, regardless of age of onset, gender, and history of childhood sexual abuse.

## Method

### Data source

All public hospital services in Hong Kong are managed by the Hospital Authority (HA). This prospective study followed a cohort of patients suffering from childhood maltreatment between January 1, 2000 and December 31, 2010 over a median period of 12.47 (range: 8.09–18.08) [16]. Eligible patients were identified from the Hong Kong Clinical Data Analysis and Reporting System (CDARS) which is a database developed by the HA for research purpose. Data from the CDARS are frequently used in local and cross-regional epidemiological research [17, 18]. The authors assert that all procedures contributing to this work comply with the ethical standards of the relevant national and institutional committees on human experimentation and with the Helsinki Declaration of 1975, as revised in 2008. All procedures involving human subjects/patients were approved by the Institutional Review Board of the University of Hong Kong/Hospital Authority Hong Kong Western Cluster (UW 18–442). Moreover, all patient records in the CDARS database are de-identified and involve no risk of patient identification. Thus, informed consent was waived by the board.

### Study sample

At each public hospital, professionals from different disciplines work together to carry out the investigation of patients admitted with suspicion for child maltreatment. Cases confirmed as established child maltreatment are assigned with an International Classification of Diseases, Ninth Revision, Clinical Modification (ICD-9-CM) code. In this study, the exposed group comprised patients aged 0 to 19 years who had been admitted with a first-listed record of maltreatment episode defined by the ICD codes (995.5, 955.8, and E967.0–E967.9) from January 1, 2000 to December 31, 2010 in the CDARS database. For each exposed patient, the month of first assignment of child maltreatment code was defined as the first follow-up month.

We randomly selected at most five comparison patients per exposed patient from a subsample of individuals of the same gender and age who were admitted into the same hospital in the same calendar year and month but had no records of maltreatment (995.5, 955.8 or E967.0–E967.7), congenital anomalies (740–759), severe chronic illnesses, or an index hospital stay >14 days at any point in the CDARS database. The admission month was defined according to the case’s month of first-recorded maltreatment episode. For all comparison patients, the matched admission month was the first follow-up month.

### Procedure

The exposed and unexposed groups were followed from the month of first-recorded maltreatment episode (or matched admission month for comparison patients) until the first record of psychiatric diagnosis or January 31, 2019, whichever came first. The ICD codes used to identify psychiatric diagnosis were 290–316. The eight psychiatric disorders of interest were: psychosis (295 and 297–298), depressive disorder (296.2–296.3, 309.0–309.1, 311, and 313.1), bipolar disorder (296.0–296.1 and 296.4–296.8), anxiety disorder (300.0–300.9, 308.0–308.9, 309.2, 313.0, and 313.2), conduct disorder/oppositional defiant disorder (ODD) (312.0–312.9, and 318.81), attention deficit hyperactivity disorder (ADHD) (314), personality disorders (301), and suicide and self-inflicted injury (950–959). We also stratified the diagnostic groups (exposed and unexposed patients) into subgroups by age of onset (young children, <6 years; and older children, ≥6 years), gender (male and female), and history of childhood sexual abuse (yes and no).

### Data analysis

The association between childhood maltreatment and risk of psychiatric disorder was analyzed using Cox proportional hazard model. The strength of association was expressed as hazard ratio (HR). Patients’ time-to-event was defined as the difference between the month of index visit (i.e., first maltreatment visit for exposed patients and matched non-maltreatment visit for comparison patients) and the month of psychiatric diagnosis or month of censoring. Patients with any psychiatric diagnosis of interest prior to the index visit were excluded. Owing to the mild differences in the matching variables between the exposed group and the unexposed group, these variables including age at index visit, sex, and status of receiving Comprehensive Social Security Assistance (CSSA) (a major form of government financial subsidy for disadvantaged families in Hong Kong which was used as a proxy for socioeconomic status in this study) were adjusted in the Cox model. To examine differences between subgroups defined by history of childhood sexual abuse (yes/no), age of onset (older/young age), and gender (male/female), ratio of HR was computed as HR_subgroup_/HR_comparison subgroup_, with a ratio greater than 1 indicating that the association between childhood maltreatment and later psychiatric disorders for the numerator subgroup was stronger than that for the denominator subgroup. A one-year landmark analysis which excludes patients diagnosed with the psychiatric disorder of interest in the first follow-up year was conducted to examine potential reverse causation (e.g. undiagnosed mental health problems may cause first-time child maltreatment). Proportional hazard assumptions were checked using Schoenfeld residuals. Data were cleaned and analyzed using statistical software R (version 4.0.2) [19]. A *p*-value of less than 0.05 was deemed statistically significant.

## Results

There were 7473 children with records of A&E attendance and/or hospitalization for childhood maltreatment during 2001–2010, whereas the matched comparison group consisted of 26,834 patients with no recorded maltreatment episode in the same period (Table 1). 26.15% of individuals with a history of childhood maltreatment experienced their first-time maltreatment episode before 6 years of age. Notably, 9.49% had a substantiated report of being sexually abused during childhood. A larger proportion of individuals with a history of childhood maltreatment (21.66%) were receiving CSSA on the date of hospital admission compared to those non-exposed individuals (19.61%). The exposed and unexposed groups were otherwise similar on age of onset and gender.

**Table 1:** Subject characteristics.

	Overall (*n* = 34,307)	Exposure to childhood maltreatment during 2001–2010		
		Yes (*n* = 7,473)	No (*n* = 26,834)	*p*-value
Age of onset, mean(SD)	8.57 (4.69)	8.71 (4.63)	8.54 (4.70)	0.006
Age of onset, *n* (%)				0.002
<6	9461 (27.58)	1954 (26.15)	7507 (27.98)	
≥6	24846 (72.42)	5519 (73.85)	19327 (72.02)	
Gender, *n* (%)				0.16
Male	16548 (48.24)	3550 (47.50)	12998 (48.44)	
Female	17759 (51.76)	3923 (52.50)	13836 (51.56)	
Receiving CSSA on the date of admission, *n* (%)				<0.001
Yes	6882 (20.06)	1619 (21.66)	5263 (19.61)	
No	27425 (79.94)	5854 (78.34)	21571 (80.39)	
History of childhood sexual abuse, *n* (%)	709 (2.07)	709 (9.49)	-	-

*CSSA* Comprehensive Social Security Assistance

Cox regression models (Table 2) revealed that childhood maltreatment exposure was significantly associated with an increased risk for both overall (HR, 4.12, 95% confidence interval [CI] [3.75, 4.53]) and specific psychiatric disorders (conduct disorder/ODD: HR, 11.37, 95% CI [7.43, 17.38]; ADHD: HR, 10.62, 95% CI [8.35, 13.50]; personality disorders: HR, 6.03, 95% CI [4.38, 8.30]; suicide and self-inflicted injury: HR, 4.33, 95% CI [3.57, 5.25]; anxiety disorder: HR, 3.88, 95% CI [3.23, 4.66]; psychosis: HR, 3.60, 95% CI [2.69, 4.84]; depressive disorder: HR, 3.48, 95% CI [2.65, 4.58]; bipolar disorder: HR, 2.08, 95% CI [1.16, 3.71]). After excluding events in the first follow-up year, all these HRs, though particularly that for ADHD, were attenuated but remained significant.

**Table 2:** Associations of childhood maltreatment with overall and specific psychiatric disorders.

	All events				Excluding events in the first follow-up year			
	*N*	Event	HR (95% CI)	*p*-value	*N*	Event	HR (95% CI)	*p*-value
Overall psychiatric disorders	33887	1736	4.12 (3.75, 4.53)	<0.001	33461	1310	3.13 (2.80, 3.49)	<0.001
Specific psychiatric disorders								
Anxiety disorder	34259	462	3.88 (3.23, 4.66)	<0.001	34197	400	3.42 (2.81, 4.16)	<0.001
Attention deficit hyperactivity disorder	34261	350	10.62 (8.35, 13.50)	<0.001	34127	216	7.28 (5.49, 9.65)	<0.001
Bipolar disorder	34301	49	2.08 (1.16, 3.71)	0.010	34297	45	1.98 (1.08, 3.65)	0.030
Conduct disorder / oppositional defiant disorder	34287	117	11.37 (7.43, 17.38)	<0.001	34239	69	10.99 (6.36, 19.01)	<0.001
Depressive disorder	34292	207	3.48 (2.65, 4.58)	<0.001	34276	191	3.07 (2.31, 4.08)	<0.001
Personality disorders	34291	161	6.03 (4.38, 8.30)	<0.001	34262	132	5.36 (3.78, 7.59)	<0.001
Psychosis	34291	178	3.60 (2.69, 4.84)	<0.001	34279	166	3.61 (2.66, 4.90)	<0.001
Suicide and self-inflicted injury	34256	416	4.33 (3.57, 5.25)	<0.001	34201	361	3.84 (3.13, 4.72)	<0.001

Adjusted for age, sex, and status of receiving CSSA.

Table 3a–c show the results of comparative analysis of childhood maltreatment effect on later psychiatric disorders by age of onset, gender, and history of childhood sexual abuse. As seen in Table 3a, the HR for overall psychiatric disorders increased by 34% in the subgroup with onset at age ≥6 years compared to the subgroup with onset at age <6 years. Notably, ADHD diagnosis showed a stronger association with childhood maltreatment exposure when the event occurred at older ages (ratio of HR, 2.37, 95% CI [1.62, 3.44]), but these differences by age of onset became trivial and non-significant after excluding events in the first follow-up year. The same analyses were repeated with different ages of onset. The result patterns for ages ≥5 vs <5 (Table S1) and for ages ≥10 vs <10 (Table S2) remained largely unchanged. On the other hand, after excluding events in the first follow-up year, the HR for ADHD diagnosis decreased by 61% in males compared to females (ratio of HR, 0.39, 95% CI [0.20, 0.74]) (Table 3b). When stratified by history of childhood sexual history (Table 3c), the HR was significantly higher among individuals with a history of childhood sexual abuse than those without for conduct disorder/ODD (ratio of HR, 2.05, 95% CI [1.13, 3.72]) and depressive disorder (ratio of HR, 1.89, 95% CI [1.16, 3.07]). However, only the ratio of HR for conduct disorder/ODD remained significant after excluding events in the first follow-up year (ratio of HR, 3.09, 95% CI [1.13, 3.72]).

**Table 3:** a. Comparison of child maltreatment effect on later psychiatric disorders by age of onset (≥6 vs <6). b. Comparison of child maltreatment effect on later psychiatric disorders by gender (male vs female). c. Comparison of child maltreatment effect on later psychiatric disorders by history of childhood sexual abuse (yes vs no).

	All events		Excluding events in the first follow-up year	
	Ratio of HR (95% CI)	*p*-value	Ratio of HR (95% CI)	*p*-value
Overall psychiatric disorders	1.34 (1.08, 1.66)	0.007	1.03 (0.81, 1.32)	0.78
Specific psychiatric disorders				
Anxiety disorder	1.18 (0.77, 1.80)	0.45	1.10 (0.71, 1.71)	0.67
Attention deficit hyperactivity disorder	2.37 (1.62, 3.44)	<0.001	1.38 (0.88, 2.16)	0.16
Bipolar disorder	1.46 (0.17, 12.44)	0.73	1.65 (0.19, 14.11)	0.65
Conduct disorder / oppositional defiant disorder	1.14 (0.54, 2.41)	0.73	0.79 (0.34, 1.88)	0.60
Depressive disorder	1.70 (0.69, 4.17)	0.25	1.58 (0.64, 3.89)	0.32
Personality disorders	1.31 (0.64, 2.68)	0.46	1.41 (0.66, 2.98)	0.38
Psychosis	0.82 (0.37, 1.80)	0.61	0.85 (0.38, 1.88)	0.69
Suicide and self-inflicted injury	1.52 (0.80, 2.89)	0.20	1.65 (0.82, 3.30)	0.16
Overall psychiatric disorders	1.14 (0.95, 1.38)	0.16	1.01 (0.81, 1.26)	0.94
Specific psychiatric disorders				
Anxiety disorder	0.79 (0.54, 1.15)	0.22	0.93 (0.62, 1.39)	0.71
Attention deficit hyperactivity disorder	0.62 (0.36, 1.06)	0.08	0.39 (0.20, 0.74)	0.004
Bipolar disorder	0.80 (0.25, 2.61)	0.71	0.76 (0.22, 2.67)	0.67
Conduct disorder / oppositional defiant disorder	1.22 (0.52, 2.85)	0.65	0.78 (0.26, 2.37)	0.67
Depressive disorder	1.07 (0.56, 2.06)	0.84	1.19 (0.61, 2.33)	0.61
Personality disorders	0.56 (0.28, 1.09)	0.09	0.62 (0.30, 1.28)	0.20
Psychosis	1.09 (0.60, 1.99)	0.78	1.04 (0.56, 1.94)	0.91
Suicide and self-inflicted injury	0.88 (0.57, 1.34)	0.54	0.87 (0.56, 1.36)	0.55
Overall psychiatric disorders	1.13 (0.91, 1.40)	0.28	1.18 (0.90, 1.53)	0.23
Specific psychiatric disorders				
Anxiety disorder	1.27 (0.87, 1.85)	0.22	0.92 (0.57, 1.49)	0.74
Attention deficit hyperactivity disorder	0.81 (0.47, 1.37)	0.43	1.08 (0.57, 2.02)	0.82
Bipolar disorder	0.55 (0.07, 4.16)	0.56	0.62 (0.08, 4.73)	0.64
Conduct disorder / oppositional defiant disorder	2.05 (1.13, 3.72)	0.02	3.09 (1.53, 6.25)	0.002
Depressive disorder	1.89 (1.16, 3.07)	0.01	1.64 (0.95, 2.83)	0.08
Personality disorders	1.62 (0.95, 2.75)	0.08	1.65 (0.90, 3.03)	0.11
Psychosis	1.28 (0.67, 2.43)	0.45	1.39 (0.73, 2.64)	0.32
Suicide and self-inflicted injury	1.25 (0.85, 1.83)	0.26	1.15 (0.74, 1.79)	0.54

Adjusted for age, sex, and status of receiving CSSA.

## Discussion

This study analyzed hospital administrative records of psychiatric diagnoses dating between 2001 and 2019 of 7473 patients aged below 19 who had experienced childhood maltreatment and compared them to the records of 26,834 patients who had not. We found that substantiated childhood maltreatment exposure was associated with a four-fold increase in the risk of subsequent diagnosis of psychiatric disorders. Although Axis I disorders such as depressive and anxiety disorders are frequently studied in childhood trauma research [20, 21], very few work has been done to quantify the strength of association between childhood maltreatment and Axis II personality disorders. This study, with robust features of matched cohort design and landmark analysis method, expands the existing body of literature by demonstrating an increased vulnerability to Axis I and II disorders following childhood maltreatment in a large sample of patients with ascertained medical records. Another noteworthy feature of this study is the comparison of childhood maltreatment effect across several types of psychiatric disorders. Results of the Cox regression analysis showed that maltreated children were 11 times more likely than their counterparts to develop conduct disorder/ODD, followed by ADHD, personality disorders, and suicide and self-inflicted injury. The associations did not vary significantly by gender and age of onset. Our findings are consistent with prior reports [1, 10], demonstrating that childhood maltreatment is a risk factor for many psychiatric disorders in western and non-western populations. To our knowledge, only one study has investigated the association between childhood maltreatment and personality diagnoses in a representative sample of US population, but their results were based on survey responses [22]. Hence, the current population-based study is the first of its kind to document the link between childhood maltreatment and personality disorders using hospital administrative data in a predominantly Chinese society. It alerts healthcare providers around the globe of the increased risk of personality disorders among child maltreatment victims and thus helps to facilitate early diagnosis and interventions to promote optimal outcomes in these affected individuals [23].

A growing body of evidence shows that heightened levels of stress in abused individuals can disrupt typical brain development after persistent exposure to stress hormones and ongoing inflammation, leading to structural and functional brain changes particularly in those areas involved in emotion and learning [24]. These structural and functional brain changes are associated with a wide range of affective and cognitive problems including deficits in attention, inhibition, and impulse control, all of which are the common features of psychiatric disorders. Consistent with previously reported associations in other populations [14, 21], the current study also found evidence for a potentially causal relationship between childhood maltreatment and later onset of depression and anxiety in the Hong Kong population. A meta-analysis of longitudinal studies published between 1990 and 2014 reported 2.03 and 2.70 as the pooled odds ratio (OR) for the association of overall maltreatment with depression and that with anxiety, respectively. Compared to these pooled estimates, the Hong Kong sample showed a slightly higher risk of anxiety and depressive disorder following childhood maltreatment which could be partially attributed to the cultural differences in coping approaches. As noted in a previous study of Chinese adolescents, the association between childhood maltreatment experiences and subsequent anxiety symptoms was significant only among students with negative coping styles [25]. In Hong Kong, a previous study of 2,496 university students found that victims of stalking or harassment were more likely to adopt negative coping strategies such as passive (e.g., use verbal ‘escape’ tactics) and avoidant strategies (e.g., blame yourself (the victim)) than proactive strategies (e.g., engage social support) [26]. This negative coping preference may then contribute to the high prevalence of internalizing disorders among maltreated victims, which could account for the higher risk of suicide and self-inflicted injury following childhood maltreatment in our sample compared to the estimates reported in other studies [27].

In addition to cognitive factors, interpersonal factors such as parenting may also be implicated in the development of psychiatric disorders. The link between childhood maltreatment and insecure attachment styles has been well documented [28]. Previous research also showed that children with insecure attachments tend to have low self-esteem and dysfunctional attitudes and negative attributional style, which are predictive of later anxiety and depressive symptoms [29]. In addition, our landmark analysis revealed significant gender differences in the association between childhood maltreatment and subsequent diagnosis of ADHD. While ADHD is often underdiagnosed in girls [30], research showed that ADHD symptoms could become more severe following trauma exposure [31]. Elevated signs and symptoms caused by the trauma exposure may in turn lead to the formal diagnosis of ADHD as observed among girls in the current study, although more studies are needed to confirm this speculation. Another observation of note is the reduced strength of association between childhood maltreatment and subsequent ADHD diagnosis after excluding events in the first follow-up year. The findings suggest that ADHD symptoms may have preceded maltreatment episodes particularly in older children. However, the reduced HR in our landmark analysis for the association between childhood maltreatment and subsequent ADHD diagnosis remains alarmingly high as 7.28 compared to the pooled estimates of OR = 2.39 reported in previous research [32]. These differences could be attributed to the cultural norms about parenting practices. For example, physical disciplines are acceptable in Hong Kong but have been banned in many other societies. In the absence of legislation on the use of physical publishment, family services that teach caregivers how to develop and practice positive discipline techniques should be provided to prevent child maltreatment.

The finding that individuals with a history of childhood sexual abuse were up to 3 times more likely than those without to develop conduct disorder is consistent with the broader literature on child sexual abuse and conduct disorder [33]. On the other hand, the landmark analysis revealed that the risk of depressive disorder following childhood maltreatment did not vary significantly by history of childhood sexual abuse. While the emotional consequences of childhood sexual abuse are known [34], our findings suggest that children and adolescents high in depressive symptoms could be prone to sexual abuse events. Notably, these children tend to live in a chaotic home environment characterized by low cohesion and high conflicts [35]. Evidence shows that domestic violence is associated with intrafamilial sexual abuse during childhood [36]. Children experiencing domestic violence are also at an increased risk of extrafamilial sexual assaults, owing to their relationship with sexually abusive peers and engagement in dangerous situations [37]. Hence, it is important to develop policies and interventions that reduce childhood exposure to domestic violence on one hand and raise public awareness of the benefits of early diagnosis and treatment of depressive disorder in children and adolescents on the other hand.

The present study had several strengths including large and representative sample, long follow-up period, and use of hospital administrative records. In addition, the landmark analysis method was applied to generate an unbiased estimate that reflects the time-to-event probabilities in each group conditional on whether patients were diagnosed with the psychiatric disorder of interest during the first year after the initial episode of maltreatment. Our results can be generalized to the majority of individuals with a history of childhood maltreatment in Hong Kong, as we included electronic health records from all public hospitals under the HA which admits more than 80% of patients in Hong Kong [38]. However, this study had several limitations that should be addressed in future research. First, the prevalence and risk of diseases and maltreatment could have been underestimated, since the services provided by private hospitals and outpatient clinics were not included, and some affected individuals in the community could be under-recognized or under-diagnosed. Second, owing to the lack of codes for specific disease diagnoses for some patients, we cannot examine the association between history of childhood maltreatment and different types of personality disorders. Lastly, hospital information on types and severity of child maltreatment was incomplete. Hence, we cannot examine the potential variations in the risk of psychiatric disorder following different subtypes of child maltreatment.

## Conclusion

Individuals with a history of childhood maltreatment have a four-fold higher risk of subsequent psychiatric disorder compared to their counterparts in Hong Kong. This research reinforces earlier observations that childhood maltreatment exposure is associated with later diagnosis of Axis I disorders. It also extends the literature by demonstrating the association between childhood maltreatment exposure and Axis II personality disorders as well as the reciprocal influences between maltreatment episodes and mental health problems. The findings emphasize the provision of integrated care encompassing medical and psychological treatment within the primary health care setting to address the long-term problems and complex needs of child maltreatment victims.

## Supplementary information

Supplementary tables

## Data Availability

The data that support the findings of this study are available on request from the corresponding author.
