# Supplementary material for: Associations between childhood maltreatment and psychiatric disorders: analysis from electronic health records in Hong Kong
Source: Transl Psychiatry. 2022 Jun 6;12:231. doi: 10.1038/s41398-022-01986-y (PMC9170694; doi:10.1038/s41398-022-01986-y)
Supplement: Supplementary file 1 — Supplementary tables [file 41398_2022_1986_MOESM1_ESM.docx]

Table S1. Comparison of child maltreatment effect on later psychiatric disorders by age of onset (≥5 vs <5)

|  | **All events** | | **Excluding events in the first follow-up year** | | |  |
| --- | --- | --- | --- | --- | --- | --- |
|  | **Ratio of HR (95% CI)** | **p-value** | | **Ratio of HR (95% CI)** | **p-value** | |
| Overall psychiatric disorders | 1.27 (1.01, 1.59) | 0.04 | | 1.02 (0.79, 1.32) | 0.90 | |
| Specific psychiatric disorders |  |  | |  |  | |
| Anxiety disorder | 1.64 (0.99, 2.72) | 0.055 | | 1.62 (0.96, 2.73) | 0.07 | |
| Attention deficit hyperactivity disorder | 2.37 (1.60, 3.51) | < 0.001 | | 1.46 (0.93, 2.30) | 0.10 | |
| Bipolar disorder | 1.00 (0.12, 8.62) | 1.00 | | 1.14 (0.13, 9.90) | 0.90 | |
| Conduct disorder / oppositional defiant disorder | 1.64 (0.70, 3.86) | 0.26 | | 1.39 (0.54, 3.54) | 0.49 | |
| Depressive disorder | 1.90 (0.65, 5.50) | 0.24 | | 1.78 (0.61, 5.19) | 0.29 | |
| Personality disorders | 2.34 (0.94, 5.78) | 0.07 | | 2.88 (1.08, 7.73) | 0.04 | |
| Psychosis | 1.21 (0.45, 3.25) | 0.70 | | 1.28 (0.48, 3.43) | 0.63 | |
| Suicide and self-inflicted injury | 1.46 (0.71, 3.00) | 0.30 | | 1.72 (0.77, 3.85) | 0.19 | |

Adjusted for age, sex, and status of receiving CSSA.

Table S2. Comparison of child maltreatment effect on later psychiatric disorders by age of onset (≥10 vs <10)

|  | **All events** | | **Excluding events in the first follow-up year** | | |  |
| --- | --- | --- | --- | --- | --- | --- |
|  | **Ratio of HR (95% CI)** | **p-value** | | **Ratio of HR (95% CI)** | **p-value** | |
| Overall psychiatric disorders | 1.12 (0.95, 1.32) | 0.18 | | 1.18 (0.97, 1.43) | 0.11 | |
| Specific psychiatric disorders |  |  | |  |  | |
| Anxiety disorder | 1.66 (1.19, 2.32) | 0.003 | | 1.41 (0.99, 2.01) | 0.06 | |
| Attention deficit hyperactivity disorder | 0.80 (0.56, 1.15) | 0.23 | | 1.11 (0.68, 1.81) | 0.67 | |
| Bipolar disorder | 0.91 (0.28, 3.00) | 0.88 | | 0.92 (0.27, 3.09) | 0.89 | |
| Conduct disorder / oppositional defiant disorder | 1.22 (0.66, 2.27) | 0.52 | | 1.37 (0.62, 3.02) | 0.44 | |
| Depressive disorder | 1.75 (0.98, 3.13) | 0.06 | | 1.51 (0.84, 2.72) | 0.17 | |
| Personality disorders | 1.60 (0.92, 2.78) | 0.10 | | 1.31 (0.73, 2.38) | 0.37 | |
| Psychosis | 1.11 (0.63, 1.98) | 0.71 | | 1.10 (0.61, 1.96) | 0.76 | |
| Suicide and self-inflicted injury | 1.16 (0.78, 1.70) | 0.46 | | 1.08 (0.72, 1.62) | 0.72 | |

Adjusted for age, sex, and status of receiving CSSA.
